# Oxidative Stress in the Anterior Ocular Diseases: Diagnostic and Treatment

**DOI:** 10.3390/biomedicines11020292

**Published:** 2023-01-20

**Authors:** Azza Dammak, Cristina Pastrana, Alba Martin-Gil, Carlos Carpena-Torres, Assumpta Peral Cerda, Mirjam Simovart, Pilar Alarma, Fernando Huete-Toral, Gonzalo Carracedo

**Affiliations:** Ocupharm Group Research, Faculty of Optic and Optometry, Universidad Complutense de Madrid, 28037 Madrid, Spain

**Keywords:** oxidative stress, dry eye, keratoconus, uveitis, conjunctiva, cataract

## Abstract

The eye is a metabolically active structure, constantly exposed to solar radiations making its structure vulnerable to the high burden of reactive oxygen species (ROS), presenting many molecular interactions. The biomolecular cascade modification is caused especially in diseases of the ocular surface, cornea, conjunctiva, uvea, and lens. In fact, the injury in the anterior segment of the eye takes its origin from the perturbation of the pro-oxidant/antioxidant balance and leads to increased oxidative damage, especially when the first line of antioxidant defence weakens with age. Furthermore, oxidative stress is related to mitochondrial dysfunction, DNA damage, lipid peroxidation, protein modification, apoptosis, and inflammation, which are involved in anterior ocular disease progression such as dry eye, keratoconus, uveitis, and cataract. The different pathologies are interconnected through various mechanisms such as inflammation, oxidative stress making the diagnostics more relevant in early stages. The end point of the molecular pathway is the release of different antioxidant biomarkers offering the potential of predictive diagnostics of the pathology. In this review, we have analysed the oxidative stress and inflammatory processes in the front of the eye to provide a better understanding of the pathomechanism, the importance of biomarkers for the diagnosis of eye diseases, and the recent treatment of anterior ocular diseases.

## 1. Introduction

The anterior segment of the eye involves the ocular structures from the anterior part of the cornea to the posterior part of the lens, including the trabecular meshwork (TM) and aqueous humor [1]. The ocular disorders related to the anterior segment of the eye, including dry eye, keratoconus, conjunctiva, uveitis, and cataract, share similar characteristics that facilitate their diagnosis. Moreover, these diseases present a complex pathophysiology related to oxidative stress, tissue damage, and inflammatory pathway.

### 1.1. Oxidative Stress in the Anterior Segment of the Eye

Oxidative stress is described as a process of excessive generation of free radicals such as reactive oxygen and nitrogen species (ROS/RNS) by mitochondrial metabolism and decreased activity of the protective antioxidants, leading to an imbalance in the system. It contributes to the increased occurrence of redox reactions, damaging numerous cellular structures, and therefore can be related to various pathological conditions [2], including several ocular diseases [3]. When generated at moderate concentrations during cellular respiration, the free radicals are involved in essential signaling processes and the immune defense system, released by phagocytes against pathogens. However, when the reactive species are not neutralized sufficiently by the antioxidants, oxidative stress arises, leading to various conditions in the cell, such as membrane lipid peroxidation, oxidative DNA lesions, and protein modifications, causing enzyme inactivation [4].

When it comes to ocular diseases, these cellular conditions have been found to play a significant role in the development of pathologies. Due to the external location of the eye, as well as its composition of several susceptible tissues, the ocular structures are particularly vulnerable to imbalanced oxidation [5]. 

Therefore, oxidative stress is related to numerous pathologies of the anterior segment of the eye as a result of cellular injuries generated by external sources of ROS such as exposure to artificial light and sunlight, environmental toxins, etc. [3], as well as direct contact to the atmospheric oxygen which also highly contributes to the vulnerability of the eye to oxidative damage [6]. When exposed to ultraviolet radiation (UVR), the light is mainly absorbed by the cornea and lens, thus inhibiting the UVR from reaching the retina. This, however, results in increased ROS production on the eye’s surface, possibly leading to molecular changes in the structures [7].

One of the leading free radicals formed in the process of mitochondrial respiration [2] and from exposure to UVR is H2O2 [8]. Generated by a transfer of two electrons to the molecular oxygen, hydrogen peroxide is a highly reactive species that is able to directly attack cellular proteins, lipids, and DNA, and cause indirect damage through interaction with metal ions [2]. Additionally, several other chemical species, such as superoxide anions and hydroxyl radicals, are also produced during the process of anaerobic metabolism in the cells [3]. For this reason, the anterior ocular structures possess a highly regulated antioxidant system to preserve the eye’s proper function [7].

The tear film contains various antioxidants, including ascorbic acid, tyrosine, glutathione, cysteine, and uric acid, which act to counteract the generated ROS. All these substances are also present in the cornea together with several additional antioxidants, such as ferritin, as well as superoxide dismutase (SOD) which are both essential in protection against oxidative damage from UVR [8], SOD being the main neutralizing enzyme for H2O2 [9]. Aqueous humor (AH), a liquid filling the space between the cornea and lens, has the leading antioxidative defense role against free radicals from UVR reaching the lens. Therefore, the fluid possesses numerous abundant antioxidants, such as the tear film. However, the levels of ascorbic acid are considerably more significant in the AH, while SOD is only present in small amounts compared to the tear film [7]. 

Due to increased age [10], as well as a result of secondary diseases [5] such as poor nutrition and treatment with specific medications [11], the antioxidative system is prone to changes, specifically in the cornea [6]. The activity of antioxidant enzymes has been shown to decrease considerably in aging corneas. In contrast, the action of the prooxidants remains without significant change [12], increasing the presence of oxidative stress and subsequent inflammation and making the eye more susceptible to injury and disease [1].

The pathogenesis of numerous conditions associated with the ocular surface, such as glaucoma, uveitis, dry eye, and cataract formation, has been directly linked to enhanced ROS production and reduced antioxidant defense [3]. 

### 1.2. Main Biomarkers of Oxidative Stress and Inflammation

Oxidative stress is strongly connected to inflammation as ROS activates several inflammatory pathways and can initiate the development of inflammation [13]. However, phagocytic cells release ROS/RNS and other free radicals during the inflammatory processes, further contributing to oxidative stress [14]. As the secretion of free radicals by the inflammatory cells is often excessive, additional oxidative stress and tissue damage may arise from the process [5].

Oxidative stress contributes to inflammation as ROS initiate multiple inflammatory pathways [13]. Pro-inflammatory agents such as IL-6, as well as interferon-γ and the pro-inflammatory component of bacterial cell wall lipopolysaccharide, can enhance the production of ROS [13]. H_2_O_2_ is directly modulating the activity of the transcription factor NF-kB, which induces the expression of numerous pro-inflammatory cytokines, including IL1 and IL6, as well as TNF-α [8].

Oxidative stress and inflammation are also tightly interconnected through NOD-like receptor protein 3 (NLRP3) inflammasome, which is activated by ROS from damaged mitochondria, leading to the secretion of cytokines IL-1β and IL-18 that trigger local inflammation [5]. In addition to several cytokines, 8-hydroxy-20-deoxyguanosine (8-OHdG) has also been noted as an essential biomarker of oxidative DNA damage in the TM since increased levels of 8-OHdG are related to increased intraocular pressure as well as loss of vision in glaucoma patients [9].

## 2. Mechanism of Oxidative Stress in Anterior Eye Disorders 

### 2.1. Oxidative Stress in the Ocular Surface

#### 2.1.1. Dry Eye

Dry eye disease (DED) is a multifactorial and complex disease of the tear film, eyelids, lacrimal glands, and a variety of ocular surface tissues, such as epithelial cells and goblet cells [15]. It is defined as a common painful visual disturbance with symptoms of discomfort and tear film instability [16]. The origin of DED comes from systemic inflammatory diseases, localized eye problems, or commonly used medications, for instance, chronic use of eyedrops related to glaucoma treatment [17]. However, several external factors may be associated with DED, such as environmental factors, including exposure to pollutants, ultraviolet (UV) radiation, and ozone. These external factors increase oxidative stress on the ocular surface, inflammation, and osmolarity of the tear film [16,18].

Different studies demonstrate that the incidence of dry eye increases with age [19]. On the other hand, aging is associated with increased oxidative stress [20], which is closely linked to inflammatory pathways and accompanied by the production of reactive oxygen species (ROS) [21]. In normal conditions, ROS levels are counter-balanced by physiological antioxidants; however, with age, the balance between oxidants and antioxidants is not equilibrated, leading to the overproduction of radical and non-radical species [22]. The excess of these reactant molecules plays an adverse role in the standard physiological systems as oxidative stress mediates cellular oxidative damage [23]. Continuous reduction of the antioxidant defense mechanisms and ROS overproduction can cause cellular dysfunction [24]. Moreover, hyperosmolarity plays a critical pathogenic role in DED since it induces continuous oxidative stress damage to the ocular epithelium surface. Oxidative products cause mtDNA and membrane damage from lipid peroxidation [25]. 

Nevertheless, oxidative stress is also caused by the activation of inflammatory cascade events and releases the inflammatory mediators that are considered biomarkers for the tears [26,27,28]. The level of the biomarker Interleukin- 6 in tears and conjunctiva stimulates cells’ production of ROS, prostaglandins, and other enzymes, in an environment with low antioxidant levels [27,29,30]. In this context, the secretion of increased cytokines and chemokines initiates the innate immune response and amplifies the inflammatory response [27,28].

In addition, recent studies on animal models demonstrated that reduced levels of antioxidants in tear protein generate meibomian gland orifices occlusion because of the fibrosis mechanism and infiltration with monocytes and neutrophils in the lacrimal glands due to the inflammatory pathway [31]. Therefore, the meibum lipids delivery to tear film is omitted, leading to tear instability and an aggravated form of dry eye.

In normal conditions, tear fluid contains several antioxidants to protect the ocular surface against certain radicals, such as ascorbic acid, lactoferrin, uric acid, and cysteine [32]. Moreover, the tear film covering the ocular surface is a mechanical and antimicrobial barrier [6]. However, in one mouse model study, it has been demonstrated that aqueous tear fluid and protein production are reduced because of mitochondrial oxidative damage in the lacrimal gland accompanied by elevated ROS production [33]. Consequently, the ocular surface is no longer protected in the eye’s anterior segment. Animal studies provide invaluable information about the role of oxidative stress in the cornea in various forms of dry eye disease [34]. For instance, Nakamura et al. demonstrated the relationship between the deposition of oxidative stress and corneal epithelial changes in the blink-suppressed dry eye mice model. They showed a strong correlation between the accumulation of oxidative stress and corneal epithelial alterations in the dry eye. This modification in the cornea is due to a reduction in blinking accompanied by elevated ROS production that overcomes the antioxidant capacity in the corneal epithelium in this dry eye mice model [27].

In Ref. [35], according to in-vitro studies, hyperosmolarity can engender oxidative stress in cultured primary human corneal epithelial cells (HCECs). In this environment, membrane and mitochondrial DNA damage shown by oxidative markers in membrane lipid peroxidation were proved because of increased ROS production, dysregulated levels of oxygenase and antioxidant enzymes, amplied inflammation pathways, and corneal alterations in DED [25]. Conferring to this evidence in the literature, scientists proved that oxidative stress might have both a direct and indirect effect on ocular surface health and that it plays an essential role in the pathogenesis of several forms of dry eye [35].

To conclude, normal aging and many acute and chronic diseases induce overexpression of ROS in the ocular surface. In this anterior segment of the eye, oxidative stress damages the ocular surface and plays a vital role in the mechanism of dry eye disease [35].

#### 2.1.2. Corneal Pathologies

The cornea is a significant part of the optical system, with 80% of the eye’s refractive power. It compromises avascular cells, which obtain water and nutrients from the tear fluid, aqueous humor, and blood vessels. Its structure comprises five external and internal layers: the epithelium, Bowman’s layer, stroma, the Descemet membrane, and the endothelium. Any aberration of these components alters the communication between these layers, and impacts their function and formation [36]. Type 1 collagen is the principal component of the cornea and represents about 80% of all corneal proteins [37].

The cornea is exposed to high-tension atmospheric oxygen, sunlight, Ultraviolet (UV), smocking, and toxic pollutants leading to ROS generation and, consequently, increased oxidative stress in the cornea [38], which results from the imbalance between ROS and antioxidant production, or the capacity of the corneal cells [39].

Despite its exposure to UV light and high oxygen tension, the cornea requires robust antioxidant defenses [19]. However, there are many harmful effects of Ultraviolet radiation (UVR). For instance, UV-B can provoke homolytic fission of H_2_O_2_, leading to hydroxyl radical generation. UV-A and -B can elicit DNA mutations and activate transcription factors, such as AP-1 and NF-kB, leading to the induction of collagen-degrading metalloproteinases and inflammation [40].

One important pathology of the cornea is Keratoconus (KC) disease. KC is a degenerative disorder, marked by thinning of cornea epithelium, causing corneal ectasia, with Bowman’s layer breaks and the deposition of Fleischer’s ring-iron at the basal layer of the epithelium [41]. Historically, KC has been described as a non-inflammatory disease [42]. However, numerous recent studies have related this pathology with inflammatory mediators, and oxidative stress index values subsequently increased [43] Toprak et al. were the first authors to describe this relationship [44]. In the oxidative and disordered environment in the cornea, ROS is raised, including an increase in superoxide production [45].

Moreover, antioxidant defense reduction causes keratocyte apoptosis and extracellular matrix (ECM) changes and mtDNA damage. Continuously increased ROS accelerates the process of keratocyte apoptosis, engendering the thinning and deformation or damage of keratoconus corneas [46]. Moreover, oxidative stress engenders the modification in the expression of the two structural compounds of Extracellular matrix (ECM), including collagen type XVIII/endostatin and collagen type XV, and leads to ECM remodeling and the degradation of collagen [47]. Consequently, in the main structural element of the ECM, the regulation mechanism of enzymes leads to the expression of gelatinase A and matrix metalloproteinase 2 (MMP-2) activity [48] to digest collagen IV, collagen V, fibronectin, and lamin [49,50]. Furthermore, epithelium fragmentation and stroma thinning in the KC cornea are caused by the lower amount of TIMP-1, which usually inhibits apoptosis in many cell types [51]. In the same way, prolidase, which is a manganese-dependent MMP, catalyzes the final step of collagen degradation, laying an essential role in matrix remodeling [52,53] and has been related to the physiopathology of KC as prolidase activity (PA) from plasma samples, which are significantly lower in KC patients than in healthy subjects [54]. An increased expression of collagenases (MMP1 andMMP-13), stromelysin (MMP-3), and matrilysin (MMP-7) in tears of KC patients [55] has also been reported.

Moreover, excessive ROS and RNS possibly originate from the dysfunction of CuZn superoxide dismutase (CuZn SOD). They induce collagenase and gelatinases responsible for the corneal thinning process in KC tissues [56]. 

Although each disease manifested a discrete free radical damage profile, oxidative stress appeared to play a more prominent role in KC, as evidenced by the significantly higher levels of immunoreactive MDA and nitrotyrosine. In addition, abnormal expression of several major antioxidant enzymes has also been reported in KC corneas [6].

#### 2.1.3. Conjunctival Pathologies

The conjunctiva is the thin mucous membrane that covers part of the eye’s front surface. It relies on the cornea at the limbus and the skin at the margin of the eyelids, forming, in this way, a sac between the globe and the eyelids [57]. It has a vital role in both vision and immunity. In addition, it protects the delicate structures of the eye by serving as a mechanical barrier to external substances [58]. Moreover, the conjunctiva is one of the wealthiest goblet-cell tissues in all bodies. These goblet cells secrete mucus, forming the tear film’s inner layer and helping to prevent dry eye syndrome [57]. 

In this context, the conjunctiva can be linked with a dry eye through various mechanisms such as inflammation, oxidative stress, disturbance of the tear film, and conjunctival fibrosis. Thus, normalization of the conjunctiva is essential for managing dry eye [58]. Dry eye is accompanied by inflammation with increased levels of inflammatory and T cell-related mediators such as IL-1β, IL-6, TNF-α, IL-17, and IFN-γ, noted in the conjunctiva and tear fluid of dry eye patients [59].

On the other hand, a recent study demonstrated that goblet cells in the conjunctiva are susceptible to the oxidative ocular environment. Moreover, the production of mucins and goblet cells have been implicated in immunomodulation on the ocular surface [60,61]. In this visual environment, reactive oxygen species generated from normal metabolism are overwhelmed by various antioxidant defense systems, preventing DNA, lipid, and mitochondria damage [59]. In fact, according to animal studies, mice with increased mitochondrial oxidative stress or a decrease in antioxidative pathways and altered homeostasis with aging, present a decreased expression of antioxidant enzymes and an increased expression of oxidative stress markers in conjunctiva [62,63]. In addition, elevated tear concentration of lipid oxidative stress markers correlated significantly with staining scores and inflammatory cell density in a confocal microscopy study evaluating [62]. 

Several disorders impact the conjunctiva, with different origins, that can be caused by bacteria, viruses, allergies, inflammation, or oxidative stress. Several studies have demonstrated that oxidative stress participates in pterygium, a degenerative condition characterized by fibrovascular outgrowth of conjunctiva over the cornea [64]. Ultraviolet (UV) exposure is the major environmental factor for pterygium development [65]. Accordingly, photo-oxidative stress leads to the chronic formation of reactive oxygen species and DNA damage [66,67]. 

The molecular pathogenesis of pterygium is complex. Liu et al. demonstrated that oxidative stress markers, inflammatory mediators, angiogenic factors, and diminished DNA repair enzymes are suggested to play an important role [68,69]. Furthermore, current knowledge regarding the molecular pathogenesis of pterygium is mainly based on tissue analysis. Based on this, increased nitric oxide (NO), decreased antioxidant enzymes, altered p53 protein, and increased 8-hydroxydeoxyguanosine (8-OHdG) as a significant biomarker of oxidative DNA damage were demonstrated in pterygium tissue [70,71].

The role of oxidative stress was investigated by several studies; however, these studies were based on the analysis of excised pterygium tissue [70,72,73]. 

In the conjunctiva, increased oxidative status causes increased systematic DNA damage. In this environment, antioxidant capacity shows a compensatory increase to keep systemic oxidant/antioxidant balance at a physiological level. 

### 2.2. Oxidative Stress in the Anterior Chamber

The eye’s anterior chamber is the space between the iris and the inner surface of the cornea. It is filled with aqueous humor. Glaucoma is the main pathology associated with this part of the eye [74]. According to recent studies, glaucoma has been demonstrated to be closely related to oxidative stress [75]. 

Glaucoma is an age-dependent disease, and the second leading cause of irreversible blindness worldwide, especially in the elderly population [76]. 

Oxidative stress is involved in primary open-angle glaucoma (POAG) since free radicals and oxidants cause damage in the TM tissues that are considered biological filters of the aqueous humor to reach the Schlemm canal [77,78]. According to different studies, morphological and functional abnormalities of the trabecular meshwork (TM) are closely related to glaucoma in an oxidative stress environment. Oxidative stress-induced dysfunction of TMCs can obstruct the outflow of the aqueous humor, leading to pathologically high intraocular pressure (IOP) and contributing to glaucoma [79]. Additionally, elevated intraocular pressure (IOP) from abnormal high resistance to AH drainage via the trabecular meshwork (TM) and Schlemm’s canal in glaucoma causes the progressive optic nerve head atrophy [36], progressive degeneration of retinal ganglion cells (RGCs), and visual field loss, all of which are considered as the clinical characteristics of glaucoma [80,81].

The human TM, described as the key area in glaucoma, is the tissue of the eye’s anterior chamber, hidden in the sclera corneal angle. TM is not directly exposed to light or UV radiation. However, it is the most sensitive to oxidative stress damage because it has less antioxidant defense than the cornea and the iris [82]. Consequently, TM cell impairment is caused by the imbalance of oxidants and antioxidants and increased resistance of the outflow system in the tissues between the anterior chamber and the lumen of the Schlemm canal [83,84]. 

In general, TM cells are essential for controlling the system of outflow homeostasis and IOP [65]. Aqueous humor (AH) is drained from the anterior chamber through the chamber angle tissue into the Schlemm canal and via the collector channels into the veins of the episcleral and conjunctiva. The role of the fluid flow of AH in the anterior chamber is to maintain IOP and provide oxygen and nutrients to the neovascularized organelle, such as the cornea, lens, and TM [81]. Trabecular lamellae are covered with TM cells in the outer. They are considered a resistor of juxta canalicular TM cells and the inner wall of Schlemm’s canal [80]. Many cell layers are immersed loosely and increased in extracellular matrix accumulation [85,86], leading to abnormal high resistance outflow of aqueous humor drainage via the TM under the inner wall of Schlemm’s canal and increased IOP.

Consequently, to the increased IOP, the amount and quality of the ECM proteins of the TM are changed [87]. For instance, fibronectin is increased in the ocular drainage outflow system of aging patients in different stages of POAG. Adhesion of TM cells to ECM proteins such as fibronectin, laminin, and collagen types I and IV, induce the cytoskeleton in TM cell structures to experience rearrangement and TM cell loss. This engenders TM disruption and dysfunction. These cell losses, or the alteration of the function of TM cells has been demonstrated by the elevation of oxidative stress [88].

In this context, oxidative stress can also induce biological reactions in the cytoskeletal structure of TM cells [88,89]. Recent studies have demonstrated that the relationship between the ciliary muscle and TM function changes in morphology, causing a change in TM micro skeleton, increased outflow resistance, and IOP elevation in glaucoma. Significant morphological changes are demonstrated in the anterior elastic tendons in the ciliary muscle. Consequently, to this modification, fine fibrils and compounds of ECM adhere to the elastic fiber network and connect to the inner endothelium, plus the increased thickness of the elastic network in TM of POAG. This fusion engenders a significant loss of TM cells [90].

In POAG patients, in the reduced reactive antioxidant environment [91], TM cells are in contact with higher concentrations of H_2_O_2_ which cause oxidative damage in the structural and functional components of mitochondria and other organelles in TM endothelial cells, such as mtDNA, proteins, and lipids membrane damage [82]. In recent years, increasing evidence has shown that mitochondrial injury and oxidative stress are involved in TM cell damage in glaucoma [92]. Indeed, mitochondrial complex I defects have been reported to be associated with the degradation of TM cells in POAG patients [93]. These findings indicate that glaucoma is a mitochondrial neurodegenerative disease and thus may suggest new options for glaucoma treatment [76]. 

In addition to POAG, other types of primary and secondary glaucoma are accompanied by an increase in IOP such as pseudoexfoliation syndrome (PEXS), pigmentary glaucoma that can be developed from pigment dispersion syndrome (PDS), uveitic glaucoma and neovascular glaucoma (NVG). These types of glaucoma include different pathologic mechanisms involved in the trabecular meshwork and anterior structures of the eye. 

PEXS and PDS are the two most common disorders to produce secondary POAG through trabecular meshwork blockage via different mechanisms [94]. Each is a defined clinical entity with the specific genes involved distinctly. PEXS is a systemic disease caused by defects in the extracellular matrix (ECM) [95]. The production and progressive accumulation of abnormal fibrillar extracellular material in ocular and extra tissues characterize it. Recent studies described that defective ECM remodelling and oxidative stress had been hypothesized as significant events leading to PEXS [95]. The link between OS and PEXS was investigated. Mastronikolis et al. confirmed that high malondialdehyde (MDA) levels, a marker of free radical-mediated lipid peroxidation related to oxidative stress, are found in the patients of PEXS [96]. Moreover, evidence shows that specific oxidation and glycation products could trigger the glaucoma formation associated with PEXS [97].

On the other hand, pigmentary glaucoma (PG) can develop from PDS in the presence of elevated IOP coupled with glaucomatous optic neuropathy, retinal nerve fiber thinning, and visual field defects [98]. PDS is characterized by a concave posterior iris surface rubbing against zonular fibers. These changes lead to the release of iris pigment granules dispersing throughout the anterior chamber by aqueous convection. The pigment deposition in the TM causes impairments to normal AH outflow and increased IOP leading to secondary glaucoma [94].

Moreover, glaucoma can be associated with uveitis. The pathologic mechanisms involved in uveitis glaucoma are complex. The diagnosis and treatment require a careful delineation of each physiopathology [99]. The inflammation pathway is implicated, hence the milieu of inflammatory cells, the mediators they release, and the corticoid therapy used to treat uveitis can participate in the pathogenesis of uveitis glaucoma. It has been proved that raised intraocular pressure is a common and severe complication of anterior uveitis. Consequently, the anatomic structure of the anterior chamber and angle is altered. These changes influence aqueous production and outflow and disrupt the homeostatic mechanisms of intraocular pressure control [100].

Another type of glaucoma is neovascular glaucoma (NVG). It is characterized by increased IOP due to the appearance of new vessels over the iris and iridocorneal and the proliferation of fibrovascular tissue in the anterior chamber angle [101,102]. Central retinal vein occlusion, proliferative diabetic retinopathy, and ocular ischemic syndrome are the common cause of NVG. These mechanisms lead to the closing of the iridocorneal angle [101]. NVG is related to the inflammation pathway. Recent studies characterized the aqueous levels of inflammation and ischemia-related biomarkers in retinal ischemic conditions, including NVG with stable iris neovascularization after anti-VEGF treatment and photocoagulation. They showed a significantly higher level of VEGF-A, IL-8, and EPO in the aqueous humor of NVG patients, compared to control groups with some retinal ischemic conditions [103]. However, VEGF is the most studied proangiogenic factor involved in NVG. It has been proved that VEGF and VEGF-m RNA levels significantly increase in the ischemic retina [102].

Oxidative stress in the TM engenders elevated ROS overproduction causing chronic stress in the retina and the optic nerve head and both dysfunction and atrophy. Moreover, ROS directly cause RGC loss by apoptosis and induced autophagy, as they are cytotoxic to RGCs [104]. Furthermore, ROS and oxidative stress impact optic nerve tissue remodeling since they trigger neuronal loss, disorganize laminar cribriform plates secondary to extracellular AGEs accumulation, and stimulate matrix metalloproteinases (MMPs) for the digestion of ECM.

At the hand of the oxidative stress pathway, the whole dysregulation of ocular homeostasis and neurodegeneration process in glaucoma is manifested by the impairment of RGCs, dendrites in the retina, axons in the optic nerve, and synapses in the brain [105]. In this condition, IOP and vascular dysregulations play an essential role in glaucoma neuropathy pathogenesis [106].

Finally, an imbalance between oxidation and antioxidation in the anterior chamber or any toxic substances present in aqueous humor may affect TM cell structures leading to IOP elevation, retinal damage in the optic nerve, and pathologic consequences in glaucoma development.

### 2.3. Oxidative Stress in the Iris

In tissues of the anterior Uvea, the concentration of Hydrogen peroxide H_2_O_2_ is measured between 30 and 70 µM [107]. This high concentration is due to the constant exposure of the eye to radiation, atmosphere oxygen, environmental chemicals, and physical abrasion [108]. Consequently, the exposure induces oxidative stress mechanisms in ocular tissues and leads to diseases such as uveitis. 

Several enzymes and non-enzymatic reactions are involved in neutralizing the oxidant, such as catalase 2 and glutathione peroxidase, in ocular tissues, including the iris and ciliary body. Moreover, these enzymes protect the anterior chamber tissue against oxidative stress [107]. Furthermore, it has been demonstrated that H_2_O_2_ is a stable intermediate oxidant that can diffuse across hydrophobic membranes, participating in producing more reactive species. 

However, the increased level of H_2_O_2_ overcomes the antioxidant status and disrupts homeostasis. Therefore, free radicals induce several damages associated with pathological conditions.

Opere et al. demonstrated that both H_2_O_2_ and synthetic peroxides exert pharmacological and toxicological actions in the anterior chamber in tissues, especially on smooth muscles of the iris-ciliary and sympathetic nerves [109]. In this context, the stress induced by oxygen-derived free radicals, such as H_2_O_2_ or superoxide anion, can deteriorate cells [110,111].

In addition, according to Green et al., H_2_O_2_ cause significant morphological changes in the iris and ciliary body and lowered intraocular pressure in the anterior chamber [112,113]. Moreover, H_2_O_2_ increases lipid peroxidation in iris epithelium cell membranes [113]. This can alter the release of neurotransmitters from central and peripheral neurons [114].

In mammals, Opere et al. demonstrated that peroxide exerts a pharmacological effect on sympathetic neurotransmission in the anterior uvea. In this context, an increase in sympathetic neurotransmission causes vasoconstriction in the anterior uvea and decreased perfusion of the ciliary process, reducing, in that case, the rate of aqueous humor and leading to a fall in IOP [109]. 

Relatively high concentrations of H_2_O_2_ (>100 nM) can enhance sympathetic neurotransmission in the iris-ciliary body of several mammalian species, an effect that is dependent on age [108].

Moreover, the effects of H_2_O_2_ on sympathetic neurotransmitter release from the iris- ciliary body involved the generation of reactive oxygen species [83]. To face oxidative stress damage, anterior uveal tissues can metabolize both endogenous and exogenous H2O2 and protect both nervous and muscular tissue from the harmful action of oxygen-derived free radicals [108].

### 2.4. Oxidative Stress in the Lens

The lens of the eye, also called the crystalline lens, is located behind the iris and in front of the vitreous body. It is an integral part of the eye’s anatomy since it allows the eye to focus on objects at varying distances due to its transparent biconvex and flexible structure [5]. A cataract is the primary pathology related to the lens. It is a partial or total crystalline lens [115].

A cataract is the most common cause of visual impairment worldwide, with the highest incidence in developing countries [116]. It reduces vision in 50% of individuals over 70 years of age [117]. The primary symptom is the loss of visual acuity. However, other symptoms can be found, such as a change in color vision, myopic shift, and photophobia. Their apparition depends on the anatomical distribution of the opacities, which could be cortical, nuclear, or posterior subcapsular [118]. 

Different types of cataracts exist; the most common is senile cataracts associated with aging [119]. Other origins of cataracts can be described as traumatism, ocular inflammation secondary to eye surgery, and systemic diseases such as diabetes. These potential causes can be classified into external agents such as UV-B radiation from sunlight and from an exogenous internal cause; such as when a crystalline structure leads to increased opacity and loss of vision [120,121]. Moreover, smoking, diabetes, corticosteroid use, and alcohol consumption represent essential risk factors for age-related cataract development [122,123]. However, the exact molecular mechanism of cataracts is still incompletely understood. The certainty that science proves resides in the fact that internal and external factors cause oxidative stress in the anterior segment of the eye and, consequently, DNA damage of the lens’ epithelial cells, protein, lipids, polysaccharides, and the nuclear acid of the lens [124]. Oxidative stress has been implicated in the etiopathogenesis of age-related cataracts because of the increased generation of active species of oxygen and free radicals in the lens [123,125]. Additionally, as described before, oxidative stress and inflammation are implicated in biochemical and morphological changes in the ocular tissues [36].

The production of ROS and free radicals-induced oxidative stress is considered one of the effective mechanisms of cataract pathology. This disorder is amplified with reduced endogenous antioxidants with age [126]. Consequently, the crystallin, the major protein in the lens, is oxidized [115]. Therefore, it becomes insoluble and progressively leads to lens opacification reducing the amount of incoming light and causing visual impairment [107]. Previous studies show that crystalline maintains the lens’s transparency through their short-range interactions [127]. However, the damage in the lens crystalline proteins causes their aggregation into insoluble amyloids and leads to cataracts [128].

The human lens contains three prominent protein families: alpha, beta, and gamma-crystallin. Both aA- and aB-crystallin are molecular chaperones comprising 30% of the lens’s protein content, which help maintain the solubility of other lens proteins, such as b- and g-crystallin [128,129]. However, b-crystallin comprises roughly 40% of the young human lens’s crystallin content and is highly modified throughout aging [130,131]. Therefore, they likely play a significant role in aging and cataract formation. Proteins are modified through post-translational modifications (PTMs) in lens proteins and become markers and vital causal factors in cataracts [132]. PTMs are related to lens opacification. Recent studies described PTMs isolated in proteins, isolated from old human lenses including phosphorylation, deamidation, racemization, truncation, methylation, and glycation [133].

Moreover, when proteins in the lens lose sulfhydryl (-SH) groups, they become cross-linked by non-disulfide bonds from high molecular aggregates and become insoluble, causing more opacification of the lens [107]. 

Recent studies report that exposure of the eye to constant solar radiation makes it vulnerable to the high burden of reactive oxygen species (ROS). The exposure of the cornea to the wavelength range of 300 mm (UVB) led to the formation and accumulation of tryptophan photoproducts in the center of the lens and the generation of singlet oxygen [134]. Consequently, damage to protein is caused by these oxygen products and causes the loss of transparency [107]. 

On the other hand, ROS induced by UV rays in sunlight cause an increased level of sodium and calcium ions and a decreased level of potassium and magnesium, and this imbalance leads to the alteration of intracellular ionic homeostasis in cataract lenses and, consequently, the high level of calcium degrades lens crystallin’s proteins and cause opacification of the lens. Moreover, elevated levels of H2O2 in the cataract lenses induce damage to lens gap junctions and alter intracellular communication, contributing to cataract formation [113].

In addition, oxidative stress-induced lipid peroxidation products such as 4-hydroxy-nominal (HNE) participate in the fragmentation of lens proteins and contribute to lens opacification with the increase in the concentration of end products (such as diene conjugates and lipid peroxides) of aqueous humor [107]. Furthermore, oxidative stress is caused by free radicals or oxidant productions, including lipid peroxidation, protein modification, and DNA damage, and results in cellular degeneration and neurodegeneration from damage to macromolecules [36,107].

In addition, the extensive oxidation of lens protein and lipids associated with human cataracts in older individuals has been found in control subjects of similar age. A deficiency of antioxidants and reactive oxygen scavengers may be involved in the pathogenesis of cataracts, as demonstrated in some experimental cataracts [135].

**Figure 1:**
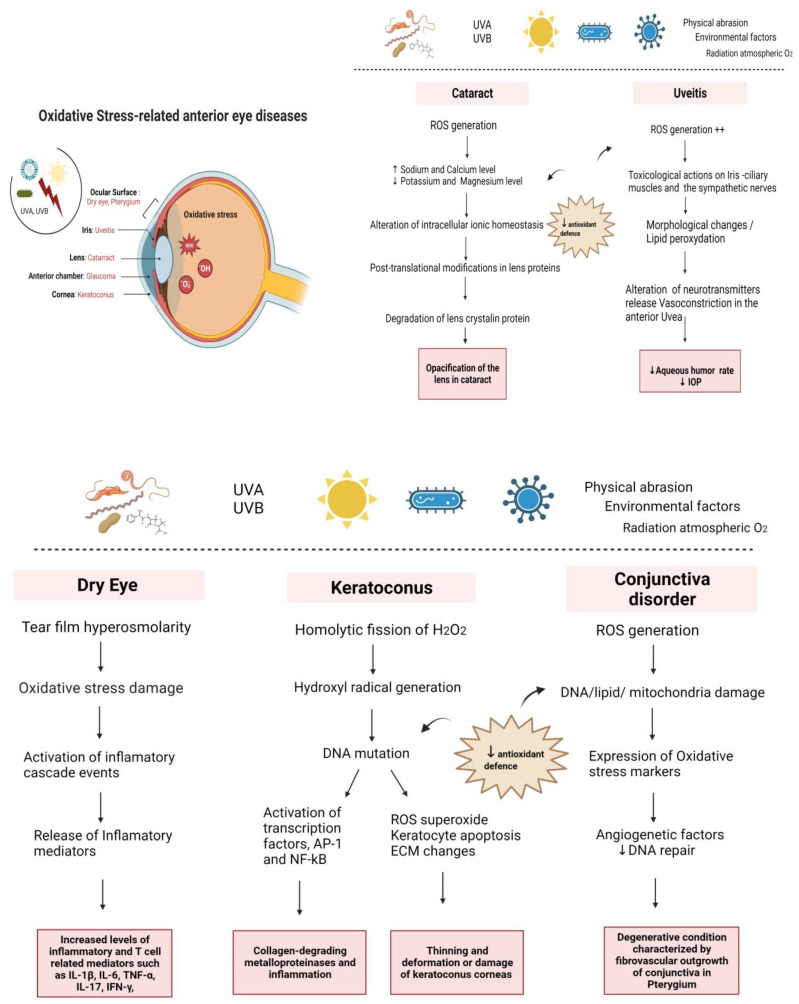
Scheme of oxidative stress role in anterior ocular diseases. UVA: Ultraviolet A, ROS: Reactive Oxygen Species, IOP: intraocular pressure, ECM: Extracellular Matrix.

In conclusion, oxidative stress induces typical changes to ocular cells and tissues, including ECM accumulation, cell dysfunction, cell death, advanced senescence, disarrangement, or rearrangement of the cytoskeleton leading to inflammatory cytokines release (Figure 1). 

## 3. Diagnostic

### 3.1. Dry Eye

The analysis of the tear film molecules opens a wide field of study for diagnosing and treating dry eye since there are specific biomolecules whose regulation is altered in dry eye cases. Many techniques are available to study tear film [136,137,138,139,140].

Inflammation in dry eye disease starts with an increase in tear film osmolarity that initiates an innate immune response [141]. The immune homeostasis of the ocular surface is controlled by lymphocytes and regulatory T cells [141] that interact with anti-inflammatory factors such as interleukin (IL)-1, transforming growth factor (TGF)-β2, and matrix protease inhibitors [141]. Cytokines and chemokines, tumor necrosis factor-alpha (TNF-α), IL-17A, IL-6, IL-8, and matrix metalloproteinase-9 (MMP-9) are the most frequently described inflammatory biomarkers [142,143]. In addition, lipid metabolism components such as secretory phospholipase A2, prostaglandin E2, arachidonic acid, docosahexaenoic acid, eicosapentaenoic acid, and leukotriene B4 have been described [142].

Regarding biomarkers, proteomic analysis of tears has recognized more than 500 proteins as biomarkers for dry eye disease [143,144,145]. Some studies have found a deficiency of lysozyme-C, lipocalin 1, lactoferrin, lysozyme proline-rich protein 4 and 3 [146], and decreased levels of annexin 5, alpha 2-glycoprotein 1, lacritin, caspase 14, proline-rich protein 3 and 4, cystatin S, cathepsin B, secretoglobin 1D1, prolactin inducible protein, and Mucin 5AC [144,147,148].

Increased levels of annexin 2, enolase 1α, albumin, nerve growth factor, clusterin, β2 microglobulin, calgranulin A (S100 A8), and B (S100 A9), cystatin SN, cathepsin S, defensins α and β, glycoprotein 340, and secretoglobin 2A [144,147,148] have also been found.

Among these dry eye biomarkers, lactoferrin and lysozyme have been considered the more accepted ones since a decrease in lactoferrin and lysozyme and an increase in albumin are related to an early inflammatory response in dry eye disease [10]. The levels of lactoferrin of <18%, lysozyme of <35%, and albumin of >15% have been reported as unique for severe dry eye disease [144].

In addition, exposure to environmental factors, chronic therapy with preserved eyedrops, inflammatory reactions, and decreased antioxidant proteins such as lactoferrin and lysozyme lead to oxidative stress on the ocular surface [16]. Late lipid peroxidation markers 4-hydroxy-2-nonenal and malondialdehyde increase in the tear film of patients with DED as indicators for oxidative damage. These molecules correlate well with ocular surface parameters such as TBUT, Schirmer’s test, and corneal sensitivity [149]. Lactoferrin, S100A proteins, superoxide dismutase, peroxidase, catalase, and mitochondrial oxidative enzymes are considered DED’s most critical antioxidant defense markers [150].

Concerning tear biomarkers for continuous use of contact lenses, it has been demonstrated that intolerance is one of the most common complications among contact lens wearers and the main reason for abandoning the contact lens [151]. The action that the contact lens and environmental factors have over the ocular surface may alter the structure and stability of the tear film, causing DED. Increased tear evaporation, hyperosmolarity, inflammation, and contact lens surface dehydration can lead to DED [138].

High levels of tear lipocalin and activation of phospholipase A2 [152,153] decreased levels of lactoferrin and lysozyme, secretoglobin 1D1, β2 microglobulin, proline-rich protein 4, and lacritin, as well as increasing the levels of protein S100A8 in soft contact lenses, secretoglobin 1 A2, albumin, nerve growth factor, and prolactin inducible protein [142,143,147,148,152,153].

### 3.2. Keratoconus 

Many ocular diseases are related to oxidative processes, so the oxidative biomarker’s presence in serum, tear film, or blood is not specific to KC diseases [154]. At present, studies of oxidative markers related to KC are conducted for a better understanding of the pathogenesis of this condition instead of finding a new diagnosis approach. In clinical practice, diagnosis of keratoconus performs analysis of corneal morphology through tomography, topography, and OCT techniques [20]. 

Nevertheless, it has been reported in numerous studies that oxidative stress biomarkers and antioxidants from the cornea or different fluids, such as tear film, aqueous humor, or blood, are dysregulated in KC. Oxidative stress markers are increased while total antioxidant capacity is decreased in several samples of KC compared to healthy subjects by 40% in the tear film.

For oxidative stress markers o indicators, monocyte/high-density lipoprotein cholesterol ratio (MHR) and neutrophil/lymphocyte ratio (NLR) from blood samples have been suggested as reliable markers for inflammatory and oxidative stress status in many diseases as KC, being higher in KC patients than in healthy subjects, approximately 50% more of MHR and 35% more of NHR [155]. In addition, 8-oxo-2’-deoxyguanosine (8-OHdG), a typical oxidative stress-induced DNA damage marker, is raised in KC corneas [156,157]. PA from serum samples, as has been described above, is significantly lower in KC patients than in healthy subjects, by about 20%, while on tear samples it is decreased by about 5%, but it is not significant [54]. 

Regarding antioxidant biomarkers, Balmus et al. have described how superoxide dismutase (SOD) enzymatic activity significantly decreases tear film from KC patients concerning healthy subjects, which could indicate a decrease in ROS neutralization and, consequently an increased cellular oxidative stress in the cornea. By contrast, the same authors reported a significant increase in glutathione peroxidase (GPx) enzymatic activity in the KC tears. Although, this discrepancy could be due to the local compensatory effect as a response to local damage induced by Cu-Zn-SOD activity in neuronal injury. Moreover, this study shows that malondialdehyde (MDA) concentrations are increased twofold in the tear film from KC patients than in healthy subjects [158], which is in agreement with previous studies that show MDA levels increased in the plasma of KC patients [159]. Catalase (CAT) is one of the most critical antioxidant enzymes in the defense mechanism against oxidative stress. CAT activity in the cornea is lowered in KC patients than in healthy subjects, and the lower activities are related to higher KC stages [160]. Similarly, it describes an apparent connection between KC diseases and thiol-disulfide homeostasis. Native and total thiol-disulfide levels in plasma are significantly lowered in KC patients than in healthy subjects, while disulfide levels, disulfide-native thiol ratios, and disulfide-total thiol ratios are significantly increased [43].

In the same way, there are other nonenzymatic molecule antioxidants in tear film and serum, such as glutathione, an antioxidant, and the most prevalent endogenous thiol-containing molecule. Glutathione is significantly lowered in KC patients’ tear films, cornea, and serum [43,161,162,163]. L-tyrosine, another nonenzymatic molecule antioxidant, has been suggested as a potential indicator of KC due to being significantly enhanced in tear film [161]. Uric acid is also a nonenzymatic molecule antioxidant presented in serum, tear, and aqueous humor samples and is raised in the tear film from KC patients [164]. Moreover, some trace elements with antioxidant activity, such as Cu, Zn, and Se, decreased their activity in serum samples from KC patients concerning healthy subjects [165,166]. Lactoferrin is a molecule involved in corneal defense against infection and has an antioxidant capacity, which is reduced in KC tear film [167].

### 3.3. Conjunctiva

The clinical signs that should be considered in the differential diagnosis of conjunctival inflammation are the symptoms, the type of discharge, the conjunctival appearance, the membranes, and the presence or absence of lymphadenopathy.

At the cellular and molecular level, different studies include biomarkers to help diagnose conjunctival disease.

Due to the conjunctiva’s primary function, maintaining the ocular surface’s homeostasis [168], it is a valuable tool for evaluating biomarkers of multiple eye disorders with minimal eye discomfort.

Therefore, the presence of biomarkers at the conjunctival level is an indicator of conjunctival pathology and other pathologies directly related to this tissue, such as dry eye disease, Sjögren Syndrome, Stevens-Johnson Syndrome, atopic keratoconjunctivitis, vernal atopic conjunctivitis, giant papillary conjunctivitis, Meibomian gland dysfunction, and pterygium or even other ocular pathologies such as glaucoma and graft versus host disease [146].

Few several markers have been identified with pathology related to the conjunctiva: Increased expression of Human Leukocyte Antigen-D-Related (HLA-DR) of the conjunctival epithelial cells has been associated with dry eye disease [169]. On the other hand, intercellular adhesion molecule 1 (ICAM-1), also known as cluster of differentiation 54 (CD54), is expressed on various cells such as endothelial cells, fibroblasts, leukocytes, keratinocytes, and epithelial cells [170]. The upregulation of ICAM-1, among other inflammatory markers, has been demonstrated in the conjunctiva of patients with Sjögren’s Syndrome [171]. In addition, it has been identified HLA-DR and ICAM-1 increased the expression of both these markers in dry eye patients [172]. HLA appears as a marker of aging processes [173], and it has been demonstrated that oxidative stress increases ICAM-1 in Lung epithelial cells [174], so the expression of these markers in inflammatory pathology of the conjunctiva could also be related to oxidative stress. 

Decrease in goblet cell density occurs in aqueous tear deficient dry eye and specific ocular surface inflammatory diseases, including Sjögren Syndrome, Stevens-Johnson Syndrome, ocular mucous membrane pemphigoid, and ocular graft versus host disease [175]. Moreover, changes in the population of goblet cells have been identified as a marker of conjunctival pathology. An additional functional pathway is identified in conjunctival epithelial homeostasis and goblet cell differentiation: the TGF beta signaling pathway. The conditional deletion of TGF beta signaling in K14-expressing cells in mice induces conjunctival epithelial hyperplasia and the expansion of conjunctival goblet cells [176]. On the other hand, the studies with null mice lacking goblet cells in the conjunctiva demonstrate the importance of the goblet cell in removing debris from the ocular surface. In response to the lack of goblet cells, the conjunctival epithelium showed increased expression of genes related to epithelial stress, keratinization, and inflammation, several of which are upregulated in the human dry eye [177].

Moreover, on the human ocular surface, it has long been shown that the reduction of goblet cells within the conjunctiva is presented in the most severe healing diseases that often result in keratinization and opacity of the cornea (keratoconjunctivitis sicca) [178]. However, interestingly, vitamin A deficiency can lead to the loss of goblet cells in the conjunctiva. Vitamin A has long been known to affect the differentiation of the mucous epithelium, and its deficiency affects the health of the ocular surface epithelium [179]. In addition, it has been demonstrated that the antioxidant activity of vitamin A, because of its structure, can autoxidize when oxygen pressure increases. Thus, it is the most effective antioxidant at low oxygen pressures that are typical of physiological levels found in tissues [180].

A significant decrease in secretory glycoprotein 5AC (MUC5AD) expression has also been observed in patients with atopic keratoconjunctivitis [181]. There are studies in which the results implicate oxidative stress in stimulating mucin synthesis in airways [182]. In this sense, studies would be needed to link oxidative stress and mucin expression in the conjunctiva. 

The increase in hyaluronic acid (HA) content in the tears of patients plays a role in the tear fluid of one of the most common conjunctivitis types, unilateral acute adenovirus conjunctivitis. The quantification of HA in the tear fluid is a rapid, sensitive, specific test. This molecule might be a biomarker candidate for acute conjunctivitis [183]. In cartilage, studies have demonstrated the action of HA to prevent mitochondrial dysfunction and mitochondria-driven apoptosis caused by oxidative stress [184]. It would be interesting to study whether the increase in HA in a patient with viral conjunctivitis could be a natural mechanism against oxidative stress caused by the virus. However, curiously, it is RNA viruses that induce oxidative stress. 

The clinical usefulness of several biomarkers has been demonstrated in patients with allergic conjunctival diseases; specifically, eosinophil cationic protein an eotaxin-2 as eosinophilic inflammation biomarkers: interleukin-4 and thymus and activation regulated chemokine (CCL17/TARC) as Th2 inflammation biomarkers; eotaxin, tumor necrosis factor-alpha and soluble IL-6 receptor as giant papillae biomarkers; and osteopontin and periostin as allergic inflammation and remodeling biomarkers [185]. 

In addition, red cell distribution width (RDW) is a widely accepted inflammatory marker. A study revealed that elevated RDW levels are significantly associated with seasonal allergic conjunctivitis in the pediatric population, which may imply a possible role of increased inflammatory status and oxidative stress in pathogenesis [186].

In any case, more research on conjunctival biomarkers related to oxidative stress is needed. It could be a valuable tool for diagnosing ocular pathologies, helping understand conjunctival pathophysiology, and trying different treatments.

### 3.4. Anterior Uveitis

The clinical characterization and the examination of the anterior pole are the primary tools for diagnosing uveitis [187]. It is essential that the diagnosis determines the etiology of the disease. For this reason, the International Uveitis Study Group established a standardized methodology for classifying uveitis based on the location of inflammation [188]. However, in many cases, the etiology of the disease remains hidden, being diagnosed as “idiopathic,” making its treatment difficult [189,190].

There are multiple triggering causes of uveitis since other systemic diseases can cause them, granulomatous, and have a local infectious or non-infectious origin [189]. The most common causes of uveitis are toxoplasma infection, sarcoidosis, HLA B27-associated uveitis, and HERPES virus infections [190].

In some cases, clinical characterization and observation will be sufficient to determine the etiology of uveitis; however, in other cases, specific tools will be necessary to confirm the etiology hypothesis. Using in vivo confocal microscopy to study keratic precipitates has proven to be a more precise and powerful tool in diagnosing this disease, and discriminating different causes based on the morphology of these precipitates [191,192]. Similarly, high-resolution computed tomography and positron emission tomography scans can be a valuable tools in diagnosing uveitis caused by tuberculosis, especially in cases that are not active in the lungs [193,194,195]. In the same way, the study of Interferon-Ƴ release assays using commercial kits (QuantiFERON-TB Gold test) can be a helpful tool when tuberculosis-associated uveitis is suspected [196]. Recently, a case has been published that establishes a similar relationship between uveitis related to sarcoidosis and interferon alpha, which suggests that new diagnostic tests will help in the etiological diagnosis of this disease [197].

The extraction and analysis of aqueous humor is a very versatile tool due to the different diagnostic techniques used. Thus, some studies demonstrate its usefulness in diagnosing herpes simplex virus, varicella-zoster virus, cytomegalovirus, and Toxoplasma gondii, using RT-PCR processes and specific antibodies [198,199,200]. The main advantage of PCR and RT-PCR systems is the use of tiny volumes and the possibility of analyzing multiple pathogens using multiplex PCR systems [201].

### 3.5. Cataracts

Inflammatory processes are mediated by inflammatory cytokines and chemokines, which are involved in the pathogenesis of diseases such as diabetes. Nevertheless, a cataract is commonly considered a non-inflammatory condition. For this reason, most studies use samples from age-related cataracts as a control to exhibit inflammatory changes associated with other ocular conditions. In the case of cataract studies, they are commonly used to compare diabetic cataracts. 

In Hamid et al. [202], high levels of IL-6, IL-8, and TNF- alpha in serum samples of patients with cataracts were incrementally more severe in diabetic patients with cataracts. Serum levels of IL-6, IL-1β, CRP, and TNF-1α [203] were higher in adult cataract patients than in non-cataract patients. E K Klein et al. [204] reported serum levels of IL-6 and intracellular adhesion molecule-1 (s-ICAM-1), a marker of vascular endothelial dysfunction, were associated with prevalent nuclear cataracts. In aqueous humor (AH), the levels of IFN-γ, IL-6, IL-13, IL-12, IL-10, IFN-α2, CCL2, CCL3, CCL4, CXCL8, CXCL9, CXCL10 were higher in patients with an age-related cataract than a congenital cataract [205]. Mitrovic et al. [206] detected higher AH levels of VEGF and IL-10 and higher serum levels of CCL2 in diabetic patients with cataracts compared to senile cataracts. Conversely, Engelbrecht et al. [117] did not find any difference in cytokines in the tear samples of cataract patients compared to those in the control, probably due to having a small sample size.

Oxidative stress is implicated in the pathogenesis of age-related cataracts [207]. Malondialdehyde (MDA), a lipid peroxidation product, is considered a biomarker. An increase in MDA serum levels in patients with senile cataracts has been reported [208,209,210,211]. Studies performed in humans and animals concluded the increment in lens MDA concentration was higher in diabetic cataracts than in senile non-diabetic cataracts [212,213]. In addition, plasma glutathione peroxidase (GPx) and hydroperoxides seem to be associated with the severity of the cataract [211]. 

Cytokine transforming growth factor-β1 (TGFβ) and MMP-9 protein expression increased in lens epithelial cells of diabetic cataract rats and along the same time compared to the control group. In an in vitro study with lens epithelial cells, Alapure et al. [214] reported that the activity of MMP-9 levels was higher in eyes with cortical cataracts. Furthermore, the level of MMP-9 activity increased gradually with age [214]. It suggests their potential role in the onset and development of diabetic cataracts.

In clinical practice, the diagnosis of cataracts is confirmed using a slit lamp, a quick and non-invasive technique. Few studies have focused on searching for new biomarkers for diagnosis. 

Studies of inflammatory and oxidative markers related to cataracts have been mainly conducted for a better understanding of the pathogenesis of this condition. Measuring the level of biomarkers related to cataracts in human samples could be a valuable new approach for diagnosis (Table 1).

## 4. Treatment

### 4.1. Dry Eye

ROS are present in the tears and conjunctiva of Sjögren’s syndrome patients, and high levels of ROS and oxidative stress have been identified in the tear film of dry eye patients [215] and animal models of dry eye [25]. ROS outside the mitochondria or in other cellular structures may be involved in inflammation which is a primary mechanism of dry eye disease [216]. Antioxidant treatments for DED can be topical, in eyedrop format, and systemic, in the form of oral supplements.

Oral lactoferrin has been evaluated as being an efficient treatment in improving tear stability and ocular surface epithelium in dry eye patients with Sjögren’s syndrome [217]. Vitamin C supplementation’s importance for ocular surface and tear health has been shown in diabetic people at risk for a dry eye [218]. Vitamin D is a potent antioxidant. Serum vitamin D levels were positively correlated to tear vitamin D levels [219], and Jin et al. found an increase in BUT and tear secretion with DED [218]. This suggests that orally supplementing the levels of systemic vitamin D may increase tear levels. Vitamin A has been used topically to protect the conjunctiva of patients with glaucoma and has improved dry eye symptoms [220].

A prospective study was an oral cocktail of an antioxidant supplement containing anthocyanosides, astaxanthin, vitamins A, C, and E, and several herbal extracts, including Cassiae semen and Ophiopogonis japonicus, which may increase tear production and improve tear film stability by reducing tear ROS [221]. 

Polyphenols are natural compounds with high efficacy in suppressing oxidative stress and inflammation. The beneficial effects of polyphenols on corneal cells are reducing the pathological processes of inflammation and oxidative stress, apoptosis, and modulation of the tear film. They showed high potential in treating neoplastic diseases and did not show significant side effects. As their bioavailability is low, application avoiding the gastrointestinal tract is recommended [222,223]. Based on the literature, topically applied polyphenols also present positive and promising outcomes against DED. Although the oral route did not show optimal absorption, a diet focused on the uptake of polyphenols could benefit patients with DED [224].

Another work reports creating a new type of antioxidant from pterostilbene (PS) and carboxyl-chitosan-modified graphene (CG) for treating DED in a mouse model. Using PS-CG on a dry eye mouse model increased the number of goblet cells and the production of mucin, and increased tear secretion [225]. 

Small lipids in eicosanoids can be either pro-inflammatory or anti-inflammatory and pro-resolution [226] and have been linked to the peroxidation of membrane lipids [227]. Nowadays, lipidomics bring the ability to assess changes in most eicosanoid species simultaneously, determining both the pro-inflammatory and anti-inflammatory contributions of eicosanoids in specific diseases leading to novel strategies for understanding and treating infection and inflammation [228].

### 4.2. Keratoconus

Regarding Keratoconus treatments, there are various methods depending on the severity and progression of the pathology. In 2015, a keratoconus management sequence flowchart was performed by an international panel of ophthalmology experts [229]. Typically, moderate cases are treated with spectacles or contact lenses, while severe cases may require surgical approaches, such as corneal cross-linking (CLX) or corneal transplantation. Interestingly, riboflavin, the molecule used during the CLX procedure, induces the antioxidant increase in corneal stromal cells and, consequently, the synthesis of extracellular matrix and reduces ROS levels [158,230].

Recently, therapies based on oxidant/antioxidant molecules have been described as a potential treatment to avoid the onset or progression of KC. In this way, lactoferrin-load contact lenses have been described as a new therapeutic approach to treat oxidative ocular surface pathologies such as KC, allowing the maintenance of lactoferrin antioxidant activity for at least 24 h [231]. In the same way, vitamin C or L-ascorbic acid (AA) has been proposed as a potential treatment for several oxidative stress-related ocular diseases due to its antioxidant effects. AA can scavenge free radicals in the presence of ROS [232]. AA serum levels could be increased by oral administration; however, it is quickly excreted by the urinary system, so eye drops are recommended for its local application. AA effect on the physiopathology of KC hasn’t been described but has been used to treat corneal epithelial defects and accelerate corneal wound healing [233,234].

### 4.3. Conjunctiva

Treatment for conjunctivitis includes many drugs, such as anti-inflammatories, antihistamines, or antibiotics, which are selected based on the etiology of inflammation [235]. Nevertheless, using vitamins, coenzyme Q10 (CoQ10), essential fatty acids, natural extracts, and other compounds with antioxidant properties has been investigated and proposed as adjuvant therapies for conjunctival inflammation.

Although no studies about the direct effect of vitamins or coQ10 in conjunctivitis were found in the scientific literature, a survey by Tredici et al. [236] on 20 professional swimmers daily exposed to chlorinated water demonstrated that the topical instillation of eye drops containing hyaluronic acid, vitamin E, and coQ10 for 60 days improved their conjunctival hyperemia and tear break-up time. Due to hyaluronic acid being a lubricant agent with regenerative properties over the ocular surface [237,238], the antioxidant properties of vitamin E and coQ10 could not be exclusively associated with improving these clinical signs.

Concerning the use of essential fatty acids, different experiments performed on animal models of conjunctivitis found that the oral supplementation of α-lipoic acid would protect the conjunctiva from oxidative stress by increasing the conjunctival levels of superoxide dismutase (SOD) and glutathione peroxidase (GSH-PX), and also the density of goblet cells, which are responsible for the mucin secretion to the tear film [239,240].

Dietary supplementation based on natural extracts has also been proposed for conjunctivitis treatment. In this sense, Destefanis et al. [241] found that administering tablets containing several natural extracts with antioxidant properties improved the conjunctival inflammation and mucus discharge in dogs affected by keratoconjunctivitis sicca. Moreover, in a rat model of allergic conjunctivitis, Unsal et al. [242] demonstrated that the topical instillation of an extract of pine bark containing procyanidins, bioflavonoids, and phenolic acids could help reduce the presence of mast cells and tumor necrosis factors (TNFs) -α and -β in the conjunctiva. In a human conjunctival epithelial cells culture under pro-inflammatory conditions, the antioxidant and anti-inflammatory properties of an extract of olive oil containing phenolic compounds was also proved [243].

Additionally, other antioxidants are presenting pharmacological activity investigated for treating conjunctivitis in animal models. N-acetylcysteine (amino acid derivative) [244,245], carvedilol (free radical scavenger) [246], SOD3 (antioxidative enzyme) [247], or skQ1 (mitochondrial-targeted antioxidant) [248] are some of these compounds studied during the last few years with promising results on decreasing oxidative stress and inflammation present in conjunctivitis.

On the other hand, recent in vitro studies explored the potential of natural extracts to prevent and treat pterygium, a conjunctival degeneration associated with an oxidative stress process [249]. Chen et al. [250] evaluated the effect of an extract of Rosmarinus officinalis on pterygium epithelial cells, resulting in an increase in cellular apoptosis associated with an amelioration of oxidative stress. In addition, López- Montemayor et al. [251] investigated the effect of an extract of sedum dendrobium on pterygium fibroblasts, resulting in a reduction of vascular endothelial growth factors (VEGFs) and connective tissue growth factors (CTGFs). 

Furthermore, in a cohort study on young adults for 8-year follow-up, sunglasses were associated with a lower conjunctival ultraviolet autofluorescence, which allows the detection of conjunctival damage related to ultraviolet radiation exposure, associated at the same time with pterygium [252].

From a practical perspective, the lack of clinical studies makes it impossible for the evidence-based prescription of these mentioned antioxidant therapies for conjunctival diseases, despite the promising studies performed in this field that require further investigation.

### 4.4. Anterior Uveitis

In the case of anterior uveitis, its treatment also depends on the etiology of the inflammation [187]. Nevertheless, the use of antioxidants has been studied as an adjuvant therapy to attenuate the inflammatory and antioxidant processes in the uvea.

Regarding vitamin therapy, several studies performed on rat models of anterior uveitis discovered that the intravenous injection of carotenoids such as astaxanthin, lutein, or fucoxanthin, among others, could improve oxidative stress and inflammation in terms of reducing the cellular infiltration in the aqueous humor and uvea but also the concentration of total proteins, prostaglandin E2 (PGE2), nitric oxide, TNF-α, interleukin 6 (IL-6), chemokine (C-C motif) ligand 2 (CCL2), and macrophage inflammatory protein 2 (MIP-2) in the aqueous humor [253,254,255,256]. In the only study found in humans, a placebo-controlled double-masked trial on 145 patients with acute anterior uveitis, the oral supplementation of vitamins C and E for two months did not improve the cellular infiltration in the anterior chamber. Still, it increased the visual acuity of these patients in a clinically relevant way compared with the placebo (0.10 logMAR or one line) [257].

Moreover, different natural extracts have been investigated for the treatment of anterior uveitis. In a rat model of anterior uveitis, Qin et al. [258] found that the intragastric administration of an extract of green tea containing catechin derivatives improved the clinical signs of the disease and reduced the number of infiltrating cells in the uvea and aqueous humor. In addition, they reported a reduction in the concentration of TNF-α, IL-6, and CCL2 in the aqueous humor and the expression of CD14 and TLR4 receptors in the uvea. For its part, Sato et al. [259] demonstrated that the oral administration of tyrosol, a natural phenolic compound of olive oil, was adequate to reduce the concentration of PGE2 and total proteins in the aqueous humor of dogs presenting anterior uveitis. On the other hand, in patients suffering from the disease, two clinical studies evaluated the effect of an extract of curcumin, rich in antioxidants, suggesting that its oral supplementation could improve the clinical signs of the disease [260] and prevent the apparition of inflammation outbreaks [261].

Finally, the effect of some antioxidant molecules has been evaluated in different animal models of anterior uveitis. Two studies performed on rats found that the intraperitoneal injection of pyrrolidine dithiocarbamate, a free radical scavenger, helped improve the disease’s clinical signs and reduced several biomarkers of inflammation and oxidative stress [262,263]. In the same rodents, Bora et al. [264] evaluated three antioxidant compounds with different mechanisms of action (CS 236, aminoguanidine, and nordihydroguaiaretic acid), confirming their efficacy in alleviating the clinical and histological signs of anterior uveitis. In addition, Chesnokova et al. [265] discovered that the topical instillation of SOD3 in a rabbit model of acute anterior uveitis was associated with a higher increase in antioxidant activity than dexamethasone but also with a higher decrease in aqueous humor leukocytes.

Again, translating results from preclinical research to clinical studies is necessary to generate evidence to satisfactorily prescribe these antioxidant therapies for anterior uveitis in clinical practice.

### 4.5. Cataracts

The primary treatment of cataracts is the extraction of the lens by surgery. Nevertheless, other alternative medicines, especially preventive ones, are being investigated. The use of antioxidants such as vitamins, polyphenols, melatonin, and natural extracts has been studied and proposed as an adjuvant treatment.

Polyphenols are commonly found in many types of food and have antioxidant properties. Quercetin has shown a protective effect against eye lens opacification. Topical [266] and oral [267] administration of quercetin in different rodent diabetic models was demonstrated to prevent or delay diabetic cataracts. This effect is due to its capacity to inhibit the enzyme aldose reductase and nonenzymatic glycation, factors associated with diabetic cataract development [268]. The administration of oral supplements of resveratrol in rats has been shown to prevent age-related cataracts by reducing lipid peroxidation and restoring antioxidant levels [269,270]. Higashi et al. [271] observed that resveratrol could delay the progression of diabetic cataracts in rats but not avoid its formation. Alvarez- Rivera et al. [272] designed a contact lens loaded with *Epalrestat*, an aldose reductase inhibitor that successfully prevented opacification of porcine crystalline lenses under hyperglycemic conditions.

N-acetylcysteine (NAC) can stimulate the synthesis of glutathione, a crucial antioxidant. Zhang et al. [273] reported that the instillation of N-acetylcysteine eye drops (0.01% and 0.05%) delayed the onset of diabetic cataracts in streptozotocin-induced diabetic rats. However, the study results could be more consistent due to NAC’s low bioavailability. For that reason, an amide derivative, N-Acetylcysteine amide (NACA), is considered an alternative therapeutic agent because of its better biocompatibility [274]. An in vitro study confirmed soft contact lenses could release both components for therapeutic reasons [275]. Administration of 1% *N*-acetylcarnosine (NAC) eyedrops improved the vision in senile-cataract patients and is proposed as a therapeutic means to prevent and treat cataracts [276,277,278,279]. Carnosine applied in the form of NAC reverses lens opacity in humans reducing lipid peroxidation products and the loss of reduced glutathione [276,280]. 

Melatonin is a neurohormone with antioxidant properties that has also been studied for cataract treatment. The injection of melatonin in streptozotocin-induced diabetic rats reduced the harmful effects of oxidative stress [281,282]. Melatonin administration (5 mg/kg) for eight weeks decreased the onset and progression of cataracts in diabetic rats by reducing the activity of aldose reductase and sorbitol [283]. Other studies demonstrated that melatonin injection in rats could protect the lens from radiation-induced cataracts [284,285,286]. 

The antioxidant role of ascorbic acid (vitamin C) and vitamin E have been studied to prevent or delay cataract formation showing similar results. Regarding vitamin C, in vitro studies with rodent lenses and in vivo animal models have demonstrated its protective effect from oxidative damage [287,288,289,290,291]. On the other hand, long-term clinical trials in humans suggest that vitamins C and E have little or no benefit for cataract treatment. A diet rich in vitamins C and E decreases the odds of cataract prevalence (OR) [292,293,294,295]. However, the intake of vitamin C and E supplements does not have any effect on cataracts [296,297,298,299,300] 49, and high doses (1 g) may increase the risk of age-related cataracts [301,302]. Age-Related Eye Disease Study (AREDS) reported that oral supplementation of vitamin C, vitamin E, lutein/zeaxanthin, beta carotene, and zinc do not significantly influence cataract progression [303].

## 5. Conclusions

The mechanism of most disorders in the anterior segment of the eye is related to oxidative stress. In the organism and normal conditions, oxidative stress is regulated by balancing the oxidant/antioxidant rate. In the case where the antioxidant defense is weakened by the increased production of ROS caused by different factors, the front of the eye is affected. Consequently, this part of the eye experiences sudden several molecular disorders such as inflammation, DNA damage, and apoptosis, leading to severe pathologies such as dry eye, cataracts, keratoconus, uveitis, and keratoconjunctivitis. 

Detecting the signature of these molecular disorders by measuring the level of relevant biomarkers for each pathology is a new approach for the successful diagnosis in the early stage of the disease. Antioxidant biomarkers and supplementation can be used for disease prevention and treatment. 

## Figures and Tables

**Table 1:** Summary of the principal biomarkers of oxidative stress and inflammation in clinical studies evaluating their presence in dry eye, keratoconus, conjunctival disorders, uveitis and cataract.

Disease	Biomarker	References
Dry Eye	TGF-β2, matrix protease inhibitors	Baudouin, C., et al. [141]
	TNF-α, IL-17A, IL-6, IL-8, MMP-9	Roy, N.S., et al. [142,143]
	Secretory phospholipase A2, prostaglandin E2, arachidonic acid, docosahexaenoic acid, eicosapentaenoic acid, leukotriene B4	Roy, N.S., et al. [142]
	Lysozyme-C, lipocalin 1, lactoferrin, lysozyme proline-rich protein 4, 3	Tamhane, M., et al. [146]
	Annexin 5, alpha 2-glycoprotein 1, lacritin, caspase 14, proline-rich protein 3 and 4, cystatin S, cathepsin B, secretoglobin 1D1 Prolactin inducible protein Mucin 5AC	Careba, I., et al. [144,147,148]
	Annexin 2, Enolase 1α, Albumin, Nerve growth factor Clusterin, β2 microglobulin, Calgranulin A (S100 A8), B (S100 A9) Cystatin SN, Cathepsin S, Defensins α and β, Glycoprotein 340, Secretoglobin 2A	Careba, I., et al. [144,147,148]
	lactoferrin of <18%, lysozyme of <35% albumin of >15%	Careba, I., et al. [144]
	4-hydroxy-2-nonenal and malondialdehyde	Choi, W., et al. [149]
	S100A Superoxide dismutase, Peroxidase, catalase,	Labbé, A., F [150]
	Secretoglobin 1D1, β2 microglobulin, proline rich protein 4, lacritin	D’Souza, S. and L. Tong, [152,153]
	Protein S100A8 secretoglobin 1 A2, albumin, nerve growth factor, prolactin inducible protein	Kramann, C., et al. [142,143,147,148,152,153]
Keratoconus	Monocyte/High-density lipoprotein cholesterol ratio (MHR) Neutrophil/lymphocyte ratio (NLR)	Katipoğlu, Z., et al. [155]
	8-OHdG	McKay, T.B., et al. [156,157]
	GPx, MDA	Balmus, I.M., et al. [158]
	CAT	Abdul-Maksoud, R.S., et al. [160]
	Native and total thiol-disulfide	Gulpamuk, B., et al. [43]
	Glutathione	Saijyothi, A.V., et al. [43,161,162,163].
	L-tyrosine	Saijyothi, A.V., et al. [161]
	Uric acid	Horwath-Winter, J., et al. [164]
	Decreased Cu, Zn and Se	Bamdad, S., N. Owji, and A. Bolkheir. [165,166]
	Lactoferrin	Balasubramanian, S.A., D.C. Pye, and M.D. Willcox [167]
Keratoconjunctivitis	TGF beta	McCauley, H.A., et al. [176]
	5AC (MUC5AD)	Dogru, M., et al. [181]
	Hyaluronic acid (HA)	Dreyfuss, J.L., et al. [183]
	Eotaxin, Tumor necrosis factor-alpha soluble IL-6 receptor	Shoji, J., et al. [185]
	Osteopontin	Shoji, J., et al. [185]
	Red cell distribution width (RDW)	Kurtul, B.E., et al. [186]
Uveitis	Interferon-Ƴ	Kato, A., M. Ishihara, and N. Mizuki [197]
Cataract	IL-6, IL-8 and TNF- α	Hamid et al. [202]
	IL-6, IL-1β, CRP, and TNF-1α	[203]
	IL-6 and s-ICAM-1	E K Klein et al. [204]
	IFN-γ, IL-6, IL-13, IL-12, IL-10, IFN-α2, CCL2, CCL3, CCL4, CXCL8, CXCL9, CXCL10	Zheng, Y., et al. [205]
	VEGF IL-10	Mitrovic et al. [206]
	MDA	Singh, S. [208,209,210,211]
	TGFβ and MMP-9	Alapure et al. [214]

## Data Availability

Not applicable.
